# Immunoglobulin Light Chain Gene Rearrangements, Receptor Editing and the Development of a Self-Tolerant Antibody Repertoire

**DOI:** 10.3389/fimmu.2018.02249

**Published:** 2018-10-08

**Authors:** Andrew M. Collins, Corey T. Watson

**Affiliations:** ^1^School of Biotechnology and Biomolecular Sciences, University of New South Wales, Sydney, NSW, Australia; ^2^Department of Biochemistry and Molecular Genetics, University of Louisville School of Medicine, Louisville, KY, United States

**Keywords:** immunoglobulin light chain, receptor editing, self-tolerance, antibody repertoire, V(D)J rearrangement, models of autoimmune disease, sub-species of the house mouse

## Abstract

Discussion of the antibody repertoire usually emphasizes diversity, but a conspicuous feature of the light chain repertoire is its lack of diversity. The diversity of reported allelic variants of germline light chain genes is also limited, even in well-studied species. In this review, the implications of this lack of diversity are considered. We explore germline and rearranged light chain genes in a variety of species, with a particular focus on human and mouse genes. The importance of the number, organization and orientation of the genes for the control of repertoire development is discussed, and we consider how primary rearrangements and receptor editing together shape the expressed light chain repertoire. The resulting repertoire is dominated by just a handful of IGKV and IGLV genes. It has been hypothesized that an important function of the light chain is to guard against self-reactivity, and the role of secondary rearrangements in this process could explain the genomic organization of the light chain genes. It could also explain why the light chain repertoire is so limited. Heavy and light chain genes may have co-evolved to ensure that suitable light chain partners are usually available for each heavy chain that forms early in B cell development. We suggest that the co-evolved loci of the house mouse often became separated during the inbreeding of laboratory mice, resulting in new pairings of loci that are derived from different sub-species of the house mouse. A resulting vulnerability to self-reactivity could explain at least some mouse models of autoimmune disease.

## Introduction

The success of the humoral arm of the adaptive immune system depends upon a diversity of antibody specificities within an individual's population of circulating B cells. This diversity is made possible by the process of gene recombination that takes place during B cell development, creating functional antibody heavy and light chain V(D)J transcripts from relatively small sets of Variable (V), Diversity (D), and Joining (J) genes. The basic processes underlying V(D)J recombination are now well understood (1, 2) and recently, thanks to advances in sequencing technologies that allow millions of different V(D)J gene rearrangements to be explored in a single individual, much has been learnt about the nature of the expressed antibody repertoire (3–8). Most repertoire studies, however, have focused upon the heavy chain repertoire. The nature of the light chain repertoire is less clear.

The diversity of the antibody repertoire is a consequence of the permutations of heavy chain V, D, and J genes, and light chain V and J genes, that are possible given the size of these sets of genes within the genome, and of the permutations of heavy and light chain pairings. This component of the overall diversity is referred to as combinatorial diversity, and is a simple reflection of the number of available heavy chain genes and κ and λ light chain genes. Additional diversity is generated during the recombination processes by imprecise joining at the V(D)J junctions. This is referred to as junctional diversity, and is principally determined by the extent to which random nucleotides are inserted between joining genes (4, 6).

In this review, we highlight important consequences for repertoire development that result from the organization of light chain genes within the mammalian genome. In particular, this organization facilitates repeated rounds of light chain gene rearrangement through the process of receptor editing. This helps to ensure that virtually all developing B cells successfully generate productive light chain rearrangements.

A number of biases and constraints are discussed which lead to substantially less diversity in the light chain repertoire than is usually calculated, and this limited diversity appears to be present in a wide range of species. We conclude that diversity is not the raison d'être of the light chain repertoire. In light of substantial evidence for a special role for light chains in autoimmune reactivity, we propose that the co-evolution of heavy and light chain genes has resulted in a limited light chain repertoire that usually serves to avoid self-reactivity. This hypothesis is explored through an examination of the generation of light chain repertoires in inbred mouse strains that are widely used in models of autoimmune disease.

## The number and organization of light chain genes within the mammalian genome

To properly understand how the heavy and light chain repertoires are generated, it is essential to have a detailed knowledge of the number of rearranging germline genes that give rise to these repertoires, and of their organization within the genome. The number of genes per species is highly variable as a result of dynamic evolutionary processes in these complex gene families. This can be appreciated by examining the phylogenetic relationships among genes within and between species, and is demonstrated in a phylogeny of functional human and mouse heavy and light chain variable genes (Figure S1). However, our understanding of the precise evolutionary histories of these genes across a larger range of species remains limited, largely due to a paucity of available genomic data.

The organization of the light chain genes is particularly complex, and quite different to that of the heavy chain genes. Heavy chain genes are found within a single gene locus (IGH), while light chain genes are generally found as two separate gene loci-the κ locus (IGK) and the λ locus (IGL). These two loci are found in virtually all mammalian species, while loci for these and other light chain variants are found in bony and even cartilaginous fish (9, 10). Such a general distribution of light chain genes between separate loci is intriguing, and suggests that this genomic organization may carry evolutionary advantages.

Within the κ chain loci of humans, mice and most other species, genes are organized in a similar fashion to the genes of the heavy chain locus (11–15). That is, a cluster of IGKV genes are found 5′ of a small number of IGKJ genes, with the IGKJ gene cluster located 5′ of a single IGKC gene. The dog genome is unusual in that half the canine IGKV genes are located upstream, and half are located downstream of the IGKJ and IGKC genes (16).

The number of functional IGKV genes varies widely between species, and this number may have some relationship to species size (Figure S1). We have argued that small species may require more germline genes because of the small burst size of the germinal center reaction in those species (17). As the number of cells responding to antigen is limited in small species, there is less chance for important higher affinity antibodies to emerge from the germinal center through the process of somatic point mutation. Critical specificities must therefore be encoded in the germline.

Sequencing of the human κ locus has identified 44 functional IGKV genes and open reading frames, which are found in two clusters that arose through segmental duplication (18, 19). An additional three functional IGKV sequences may be present in some haplotypes (20). A comparable number of functional IGKV genes (*n* = 54) was recently characterized in genomic sequences from the rhesus macaque, a commonly used non-human primate model (21). In contrast, studies of the horse genome reference sequence identified only 19 apparently functional IGKV genes (22), while 111 and 135 potentially functional IGKV genes have been found in the guinea pig and rat genome reference sequences, respectively (23, 24). Among mammalian species studied to date, the microbat (*Myotis lucifugis*) is unique in that it lacks a κ locus (25). It has been suggested that this may be part of a general simplification of immune function in a species that has met the weight to muscle challenge that is necessary for flight capability (26). This hypothesis is indirectly supported by the fact that the κ locus is also absent from the genome of chickens (25) and zebra finches (27), and it may have been lost from the genomes of all bird species.

**Figure 1:**
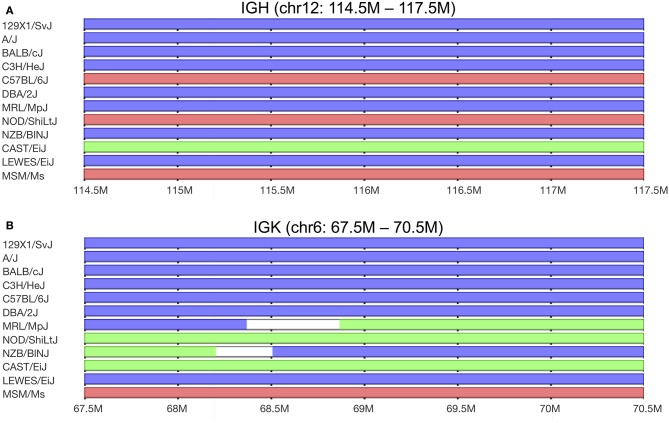
Predicted sub-specific origins of germline haplotypes found in the **(A)** immunoglobulin IGHV and **(B)** immunoglobulin IGKV loci among selected classical inbred and wild-derived mouse strains. The MRL/MpJ strain is the parental strain of the MRL/lpr strain, and although MRL/MpJ mice carry a normal Fas gene, they are also prone to autoimmunity. The lowest three strains shown are wild-derived, with genomes that are representative of the three major subspecies. *M. m. domesticus*-derived sequences are shown in blue, *M. m. musculus*-derived sequences are shown in red, and *M. m. castaneus*-derived sequences are shown in green. Data from Yang et al. (30). Graphics by Mouse Phylogeny Viewer (33).

In line with numbers reported in the small rodent species mentioned above, the genome of C57BL/6 mice carries around 101 functional IGKV genes (28). This number may, however, not be accurate for other inbred mouse strains. We recently reported that the heavy chain loci of different inbred strains of mice are derived from different sub-species of the house mouse. As a result, the C57BL/6 strain carries 99 functional IGHV genes, but the BALB/c strain carries 163 functional IGHV genes (29). We have also noted that based on available whole-genome SNP data (30), almost all inbred strains carry κ chain loci that appear to be derived from the *Mus musculus domesticus* sub-species (17). NOD/ShiLtJ mice are unusual in that they carry a *M. m. castaneus*-derived κ locus (17). Interestingly, many of the distinct κ genes of this diabetes-prone strain are identical to κ genes of the Systemic Lupus Erythematosis (SLE)-prone (NZB × NZW)F1 and MRL strains (31). SNP analysis shows that parts of the IGKV loci of NZB and MRL mice are also derived from the *M. m. castaneus* sub-species (Figure 1). This confirms an observation from a very early study of BALB/c and NZB-derived myeloma proteins. It was reported that NZB and BALB/c mice share some κ light chain sequences, but that other κ genes differ markedly between the strains (32). SNP analysis shows that the chr6:67.5m−68.2m region of the κ locus of the NZB strain is of *M. m. castaneus* origin. The chr6:68.2m−68.5m region is of uncertain origin, and the remainder of the NZB κ locus is *M. m. domesticus*-derived. Genes of *M. m. castaneus* origin are also found in the MRL strain, in the region chr6:68.8m−70.7m (see Figure 1).

The λ locus of most species investigated to date includes a set of IGLV genes that are located 5′ from a variable number of tandem cassettes, each made up of an IGLJ gene and an IGLC gene. The human locus includes as many as 38 functional IGLV genes and Open Reading Frames (18) and five functional J-C gene pairs (34). In the rhesus macaque, 47 IGLV genes are predicted to be functional based on genomic data (21), and in the pig, there are nine functional IGLV genes (35). In these species too, the IGKV genes are located 5′ of functional J-C pairs, but this organization is not invariant. 144 IGLV genes of uncertain functionality have been identified in the horse, with 110 genes being located upstream and 34 being located downstream of the IGLJ/IGLC cluster (22, 36). The locus of the mouse is also differently organized.

The C57BL/6 genome includes just three IGLV genes (Figure S1), and there has been speculation that IGLV genes might have been lost during the inbreeding of laboratory strains. In fact, diversity is lacking in wild mice of all three *Mus musculus* subspecies (37). Two of the C57BL/6 IGLV genes are associated with one functional J-C gene pair, while the third IGLV gene is associated with a second J-C pair. Lambda rearrangement in the mouse takes place within each of the two VJC units, with little or no recombination between the units (38).

The genes of both the human and mouse λ IGLV loci are all in the same transcriptional orientation as the λ J-C gene clusters (18, 39). The V, D, and J genes of the heavy chain loci of mammalian species are also found in the same orientation as their associated constant region genes (40–42). The κ chain locus of these species, on the other hand, includes many IGKV genes that are found in the opposite orientation to their associated IGKJ and IGKC genes. In the human, the orientation of the distal κ gene cluster is opposite to that of the IGKJ genes and IGKC gene, while all but the two most 3′ genes of the proximal gene cluster share their orientation with the IGKJ and IGKC genes (20). The κ locus of the mouse also includes IGKV genes in both the same and the opposite orientation to their respective IGKJ and IGKC genes (13). Such variable orientations of IGKV genes have also been reported in other species including the elephant (43), horse (22), pig (14), dog (16), and rhesus macaque (21).

In the horse, which is a species with a λ -dominant repertoire, IGLV genes are found both upstream and downstream of the λ J-C gene clusters. Many of these sequences are pseudogenes, but the few functional genes in the downstream cluster are found in the opposite orientation to that of the horse J-C genes, thereby allowing the genes to recombine (36).

**Figure 2:**
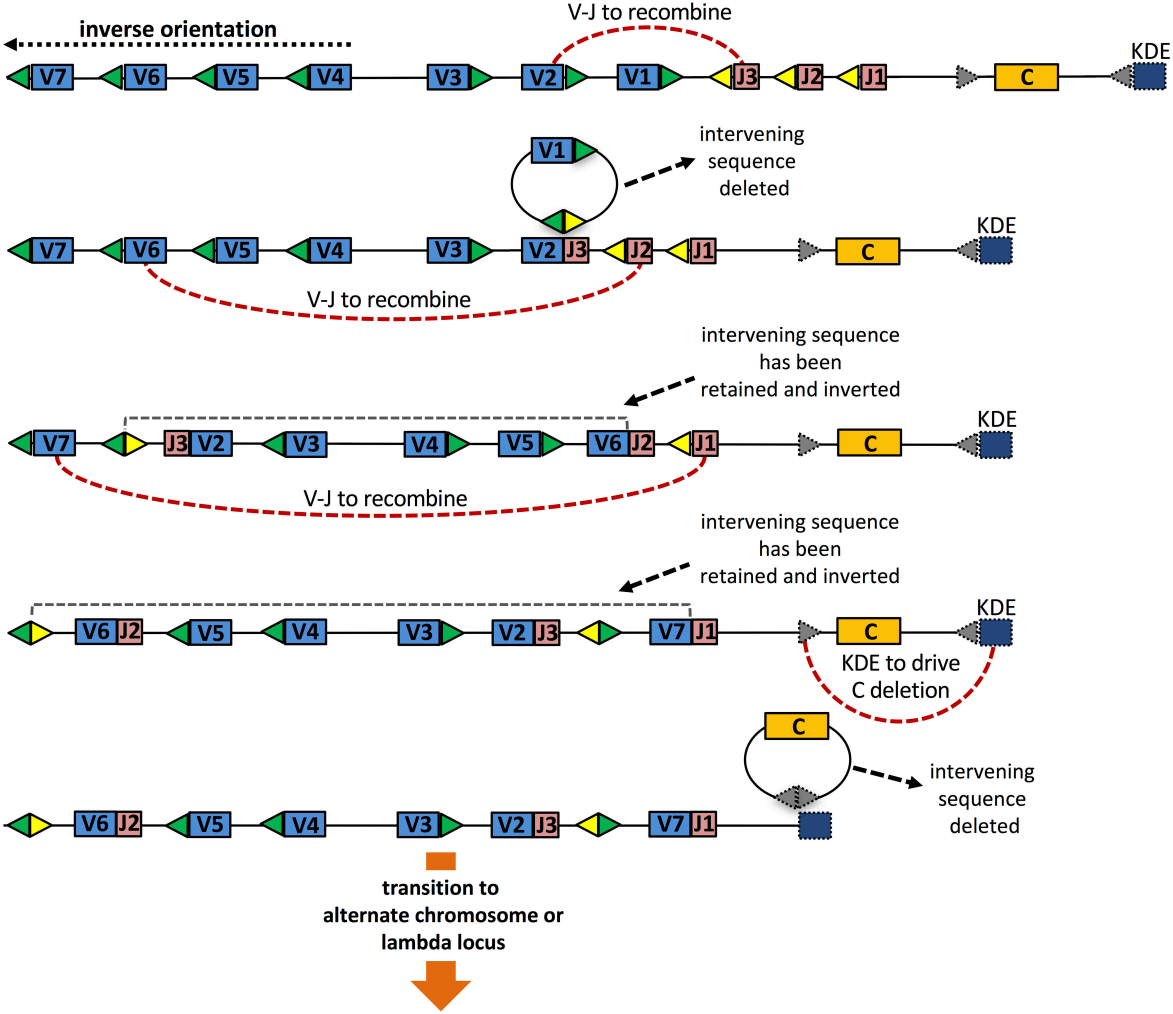
Three rounds of VJ rearrangement of the κ locus, that might result from non-productive rearrangements or rearrangements resulting in self-reactive BCR. The initial configuration of genes **(top)** shows four IGKV genes in the reverse orientation to the IGKJ and IGKC genes, and 3 IGKV genes in the orientation shared with those genes. Gene loss, as well as the changing order and orientation of genes is highlighted through successive rounds of rearrangement. Expression of the final configuration (shown in row 4), which threatens allelic inclusion and possible continuing auto-reactivity, is terminated through the action of the Kappa Deleting Element **(bottom)**.

The orientation of genes has other consequences for the generation of diversity. The opposite orientation of many IGKV genes within the murine and human κ loci means that primary rearrangements of such genes do not lead to deletion of the genes that are located between recombining IGKV and IGKJ genes (see Figure 2). This retention of genes becomes important if a rearrangement results in a non-productive chain or a self-reactive B cell receptor (BCR). In such situations, all other IGKV and IGKJ germline genes remain available for secondary rearrangements (see discussion below). In any cell that experiences such successive rounds of recombination, the order and orientation of the genes within the locus will be subject to complex changes, and this will have consequences for the repertoire that is generated by secondary rearrangements (Figure 2).

The frequencies with which different V, D, and J genes are utilized in gene rearrangements vary by many orders of magnitude. This appears to reflect, at least in part, the accessibility of genes, and their positions within the genome (28, 44, 45). Dramatic changes in the order of genes and in the distances between IGKV and IGKJ genes, arising from a primary rearrangement of genes, should therefore lead to changes to gene accessibility. This may mean that the utilization frequency of a gene can vary between primary, secondary and subsequent rearrangements. Complex changes could therefore compromise the tight regulation that otherwise appears to guide the generation of the antibody repertoire. In many species, this risk to the regulation of the repertoire may be mitigated by the action of Kappa Deleting Elements (KDE). The consecutive rearrangements that are possible within the κ locus can be terminated by KDE-mediated recombination, driving B cells to the expression of genes of the λ locus (46). This may also prevent or lead to the resolution of allelic inclusion, which can arise because of the orientation of IGKV genes within the locus (see Figure 2) (47).

**Figure 3:**
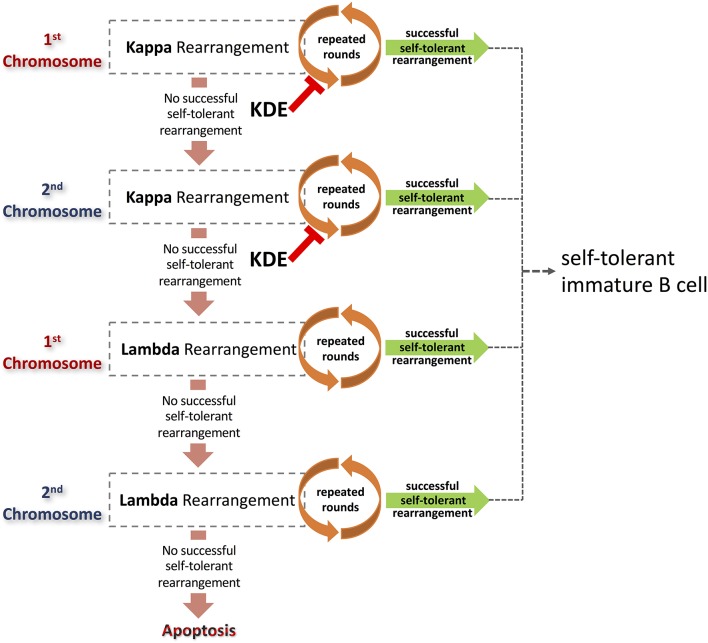
Light chain rearrangements provide multiple pathways to the production of self-tolerant B cells. Alternative pathways are a consequence of rearrangements of the light chain loci that result in non-productive or auto-reactive BCR. Beginning with rearrangements of the κ locus on the first selected chromosome, a succession of light chains can be paired with an existing heavy chain that is already expressed on the surface of the pre-B cell. Each resulting BCR is assessed for affinity to self-antigen. Cells expressing autoreactive antibodies can be rescued via further rounds of receptor editing. If repeated rounds of rearrangements of a locus fail to generate a functional, self-tolerant antibody, and the possibilities of rearrangement are exhausted, the process may continue on other chromosomes. Rounds of κ rearrangement may be prematurely curtailed by the action of the kappa deleting element. B cells unable to generate self-tolerant antibodies despite multiple rounds of receptor editing will ultimately be deleted or rendered anergic. In contrast, B cells that generate successful light chain rearrangements that result in self-tolerant antibodies will go on to develop further into immature B cells.

Kappa Deleting Elements (KDE) are located downstream of IGKC genes, and they appear to be highly conserved within the mammalian genome (48). The mouse KDE is referred to as the Recombining Segment (RS), and it is distinct but very similar to the Recombination Signal Sequences (RSS) located adjacent to the 3′ ends of the IGKV genes and the 5′ ends of the IGKJ genes (49). KDEs of all species studied are made up of conserved heptamer and nonamer sequences separated by 23 base pair spacers (48). The KDEs function by allowing recombination between the KDEs and recombining elements that contain the palindromic heptamer CACAGTG. These are located within the IGKJ-IGKC intron and in the RSS at the 3′ ends of the IGKV genes. Such recombination effectively terminates the involvement of the rearranging locus in the generation of diversity. This will drive recombination from the first to the second κ locus (i.e., on the alternate chromosome), or from the second κ locus to the λ gene-bearing chromosomes (see Figures 2, 3). It is likely that despite the conservation of this element within the κ locus, the strength of action of the elements varies between species. The preponderance of κ chains in the expressed antibody repertoire of the mouse, for example, suggests that the mouse RS usually fails to drive rearrangement to the λ locus. Instead, each murine κ locus will likely be rearranged to exhaustion, and this will prevent the overexpression of the handful of λ genes that remain in the mouse genome. The activities of RS in different sub-species of the house mouse have not been explored.

### Gene rearrangement of the light chain loci

The light chain repertoire is shaped by the order of gene rearrangement, and early studies in the mouse and human showed that rearrangement begins with the κ locus (34, 50). This may not be true for all species. It has recently been shown that the λ locus rearranges first in the fetal and neonatal pig (51, 52). Timing therefore requires further investigation, particularly in species with repertoires that are dominated by the λ light chain, for regulation of the expressed repertoire could be more difficult if the minor locus was to rearrange first. If a species had just a handful of functional κ genes, and abundant functional λ genes, initial rearrangements of the κ locus would risk over-expression of the few available IGKV genes.

In the mouse and human, if an initial κ rearrangement is unproductive or self-reactive, additional rounds of secondary rearrangement can proceed, in a process known as receptor editing (53–55). Receptor editing is usually discussed as a pathway to resolution of auto-reactivity, either in developing B cells in which self-reactivity is generated by primary rearrangements (56), or in mature antigen-selected B cells where self-reactivity may result from somatic point mutations (57). Less attention has been paid to the more general role that receptor editing plays in shaping the formation of the repertoire.

The organization of genes within the light chain loci facilitates receptor editing, and this increases the likelihood that each B cell will form an in-frame light chain rearrangement (58). As long as unrearranged V genes remain 5′ of a VJ rearrangement, and unrearranged J genes remain 3′ of the rearrangement, receptor editing can continue (see Figure 2). In the mouse, the potential of κ chain receptor editing is maximized by a bias toward rearrangement of the 5′ IGKJ1 gene (59), and this targeting results from the action of the proximal IGKJ germline transcript promoter (60).

A process of serial rearrangement of the κ chain locus may continue on one chromosome until all possibilities of recombination have been exhausted. Recombination will then proceed on the second κ chromosome (Figure 3). A failure to produce a productive, self-tolerant rearrangement on the second chromosome, after multiple rounds of rearrangement, will be followed by rearrangement of the λ loci.

The human λ locus also seems permissive of receptor editing (61), and the absence of deletional elements in the λ locus should maximize the potential of serial λ recombination in the human. This should ensure that relatively few human B cells fail to make a suitable productive light chain rearrangement that is self-tolerant when expressed in association with the cell's heavy chain rearrangement. The possibility of repeated rounds of λ rearrangement could be particularly important for the avoidance of self-reactivity, for it has been suggested that λ -bearing human B cells are less prone to self-reactivity than κ-bearing B cells (61). The λ chains of these cells may also provide stability during an ongoing immune response, for it has been shown that the codon usage of λ genes reduces the likelihood of structural changes arising from accumulating somatic point mutations (62).

### Population variation in the κ and λ gene loci

Combinatorial diversity is expanded by heterozygous gene loci, and such diversity appears to be of functional significance (63). It is therefore important that repertoire studies include a focus on alleles and gene heterozygosity. Although a few new allelic variants of human IGKV genes have recently been reported (19), the reported IGKV germline gene repertoire appears to be relatively complete (64). According to the IMGT reference directory, 26 IGKV genes have no known allelic variants, while 15 IGKV genes have only one reported variant and five have two known variants. The extent of allelic variation within the κ light chain locus could be even less than is indicated in the IMGT reference directory, for some of the reported variants are likely to be artifacts arising from sequencing errors. This is certainly the case for many reported IGHV alleles that were identified in early sequencing studies (65).

The reported human IGLV germline gene repertoire may also be relatively complete, for only five new alleles have been reported since 1997. These sequences are more varied than genes and allelic variants of the IGH and κ loci (66), but like the IGKV repertoire, there appears to be relatively little allelic variation amongst the IGLV genes. Functional and ORF allelic variants have been reported for 24 IGLV genes, but not for 15 other IGLV genes. No more than four alleles are identified in the IMGT reference directory for any IGLV gene (http://www.imgt.org/vquest/refseqh.html).

In contrast to the genes of the κ and λ loci, there is just a single functional IGHV gene (IGHV3-NL1) that lacks reported allelic variants in the IMGT reference directory or in the IgPdb database (https://cgi.cse.unsw.edu.au/~ihmmune/IgPdb/). So many common variants are known for some genes that heterozygosity in any individual is almost assured. For example there are 16 *IGHV1-69* gene sequences in the IMGT reference directory, and a further 13 alleles have been inferred from analysis of high throughput genomic and AIRR-seq data (67, 68). Although the larger number of IGHV allelic variants could reflect the greater attention that has been given to defining this set of germline genes, there is additional evidence that points to a lack of diversity in the light chain gene repertoire.

A lack of allelic variation in the human κ locus is supported by AIRR-seq studies of κ rearrangements. In a study of four individuals, involving the dominant three human IGKV gene families (IGKV1, IGKV2 and IGKV3), VJ rearrangements were seen involving between 20 and 25 genes (69). One individual was homozygous at all gene loci. In the three other individuals, heterozygosity was only seen at 1 or 2 of the IGKV loci (69). The contrast with the heavy chain locus is striking. A recent AIRR-seq study of 95 individuals explored heterozygosity at 50 heavy chain IGHV gene loci (70). Other than in three individuals from whom relatively few sequences were generated, study participants were shown to be heterozygous at between 20% and 40% of the loci. Six gene loci were heterozygous in over 50% of study participants. Only six genes that were relatively abundantly present in the datasets showed homozygosity in all individuals (70). Similar patterns of heterozygosity within IGHV coding segments have also been noted from targeted genomic sequencing data (67).

In addition to allelic variation, gene copy number variation is also enriched in the IGHV locus, relative to IGLV and IGKV. Greater than half of the known human functional/ORF IGHV genes have evidence of copy number variation (45, 67, 70–75), compared to only one and three IGLV and IGKV genes, respectively (76–78).

Additional albeit indirect evidence for an evolutionary drive to conserve rather than diversify the human κ locus comes from the similarity of the genes in the proximal and distal IGKV clusters. The large segmental duplication that gave rise to the human κ locus appears to have occurred since the divergence of the human lineage from the most recent shared ancestor with other great apes (11). There are 23 functional IGKV genes and ORFs in the proximal cluster, and 22 in the distal cluster. Eighteen paired sequences are found in both clusters, and no coding changes have evolved at eight of these paired gene loci. In addition, one sequence in each of two other sequence pairs are now non-expressible pseudogenes. Expressed variation is therefore concentrated in just 8 of the 18 sequences. Furthermore, comparisons of nucleotide variation across the entirety of the sequence comprising the large proximal and distal gene clusters reveal strong similarity. Diversity within the large segmental duplications harboring these gene clusters appears to be much lower on average (>6 fold less) than that observed within segmental duplications found in the IGHV locus (19). We have speculated that this lack of diversity in IGKV may be the result of homogenizing effects of gene conversion events between the proximal and distal regions, as such events have been explicitly documented (19). We also reported that locus-wide IGHV diversity is ~3-fold higher than IGLV diversity; in fact, IGHV diversity appears to be generally higher than the genome average (19). Earlier analyses based on limited datasets have suggested that nucleotide and amino acid substitution patterns within V segments may differ between IGHV, IGKV, and IGLV loci (79); specifically, and consistent with decreased genomic diversity in κ locus haplotypes, Schwartz and Hershberg showed that, relative to κ chain V segments, heavy and λ chain genes exhibit greater amino acid diversity in both framework and complementarity determining regions (66). Together, these data suggest contrasting evolutionary histories that have resulted in different genetic features being associated with the human heavy and light chain loci.

The κ locus of the mouse seems to display the same lack of variation that is seen in the human locus. The IGKV locus was first mapped using YACs and BACs derived largely from C57BL/6 and C3H mice (12, 80, 81), and these sequences dominate the IMGT mouse IGKV database. An alternative assembly of the mouse κ locus was later produced based upon data from the C57BL/6, A/J, 129 and DBA/2 strains (82). Each of the IGKV genes previously reported by Zachau and colleagues were mapped to this new assembly, and they were all found to have >99% identity. Not a single allelic variant was reported from this study, although it is true that their approach means that some highly similar but previously unreported polymorphisms may have been overlooked (82).

Evidence of a lack of allelic variation amongst germline genes within mouse strains also comes from analysis of the IMGT database. Studies of light chain germline genes have included a sampling of a wide variety of inbred strains, and from wild-derived *M. m. musculus* and *M. m. castaneus* mice (83–85). Yet the IMGT database includes allelic variants for just 11 functional IGKV genes, and when analysis is confined to reports from studies of strains appearing to carry *M. m. domesticus-*derived genes, variants have only been seen for 6 IGKV genes. Confirmation that the apparent lack of variation is genuine, rather than reflecting insufficient investigation of mouse light chain genes, needs to be pursued through more comprehensive surveys of variation across wild mice from each of the sub-species.

### The diversity of the expressed light chain repertoire

It is generally held that a stupendous diversity is the defining characteristic of the antibody repertories of all species. This was famously expressed by Peter Medawar as the miracle of immunology: “that a rabbit yet unborn will be able to make antibodies to an antigen not yet synthesized” (86). We have recently argued that the production of antibodies that target molecules never before seen, and unlikely to be seen, could be a costly investment for many species (17). The immune repertoires of different species may have developed varying levels of diversity in response to the quite differing evolutionary pressures faced by each species. Some of the most significant pressures may result from basic aspects of the biology of species, including their differing reproductive strategies and longevity, and especially from their varying sizes. The antibody repertoires of small species are necessarily small, and there is therefore a greater need for regulatory processes to steer the development of their repertoires toward specificities that target key pathogens (17). This may explain why in comparison to the human antibody repertoire, the murine repertoire includes more heavy chain clonotypes that are shared by many individuals of the species (6, 17, 87).

Public clonotypes may be rare in the human heavy chain repertoire, but there is a surprising lack of diversity in the human light chain repertoire, and public clonotypes account for about 60% of the human κ (69) and λ (88) light chain repertoires. This is in part a consequence of very strong biases in light chain gene usage. Six IGKV sequences dominate reported human IGK rearrangements: IGKV3-20^*^01, IGKV3-15^*^01, IGKV3-11^*^01, IGKV1-5^*^01, IGKV2-30^*^01, and IGKV1-39^*^01/IGKV1D-39^*^01 (69). The IGKV3-20^*^01 gene alone is seen in over 30% of rearrangements in some individuals (69). On the other hand, some genes are utilized at very low frequencies. In fact, amongst 22,193 rearrangements analyzed from four individuals, we saw no sequences that utilized eight reportedly functional IGKV genes (69).

Similarly, while the mouse may have over 100 available IGKV genes, just seven genes are responsible for over 40% of rearrangements, and the utilization frequencies of some IGKV genes are as low as 0.001% (28).

Biased usage of λ IGLV genes is also seen. Three IGLV genes account for more than 50% of human rearrangements, and individual IGLV genes are used at frequencies that range from 0.02 to 27% (89). In the neonatal pig, biases are even more extreme, with three IGLV genes accounting for 70% of rearrangements (51). The utilization of the four functional human IGLJ genes are also affected by biases, with frequencies varying from just 5% for IGLJ1 to almost 55% for IGLJ7 (90, 91).

The lack of D genes in light chain rearrangements limits their diversity. Diversity is further limited by the fact that relatively few nucleotides are lost from κ and λ V and J gene ends by exonuclease removals and few N nucleotides are added to the junction of the joining genes. Public human κ clonotypes have on average just 0.4 added N nucleotides, while even private clonotypes have an average of only 2.5 N additions (69). Similarly, on average, public λ VJ junctions include a single N addition, and private junctions average around two additions (88). There is even less N addition in the mouse (92), and interestingly, this is also true in the humanized mouse (88). This severely limits junctional diversity of the complementarity determining region 3 (CDR3) of light chains in the mouse. Together with the lack of combinatorial diversity, this ensures that the light chain repertoire of the mouse and human are highly constrained. In an analysis of over 250,000 mouse κ chain VJ rearrangements from 59 mice, over 90% of the sequences encoded just 1000 amino acid sequences (28). A similar number of amino acid sequences dominate the human κ chain repertoire (69).

## Light chains and the control of self-reactivity

The light chain repertoire is constrained, and there is an extensive body of research that suggests that an important factor that constrains the repertoire is the need for light chain rearrangements to minimize BCR self-reactivity. Human antibodies formed with κ chains may have a greater tendency toward self-reactivity (61), but through repeated rounds of κ rearrangement, and through similar rounds of λ rearrangement, much self-reactivity seems to be avoided. This may explain the recent observations that reduced light chain editing is associated with several autoimmune conditions in the human, including Systemic Lupus Erythematosis (SLE), type 1 diabetes (T1D), and myasthenia gravis (47, 93). This has also been observed in several mouse models of autoimmunity (47). It has also been shown that reduced KDE rearrangements can lead to dual κ and λ chain expression, through a failure to delete κ rearrangements in λ-switched cells, and this disturbance of light chain editing is associated with SLE (94).

The study of cells in which both κ and λ rearrangements are present has highlighted the fact that certain IGKV genes may be prone to self-reactivity. Biases in IGKV gene expression are seen when productive κ rearrangements are studied in λ-bearing B cells, and compared with κ rearrangements from κ-bearing cells (95). This comparison is possible because of the persistence of κ VJ rearrangements in cells that have switched to a λ light chain rearrangement as a consequence of the earlier generation of a self-reactive κ positive BCR. The biased gene expression therefore points to a tendency of some genes to mediate self-reactivity.

Some heavy chain IGHV genes are also associated with autoreactivity, and human *IGHV4-34* in particular has been implicated in anti-red blood cell autoimmune responses (96). It may be, however, that this association should be seen as resulting from a difficulty in finding a suitable light chain partner for *IGHV4-34*. The persistence of *IGHV4-34* in the human population, and its expression at relatively high frequency within the antibody repertoire, points to the value of *IGHV4-34* heavy chains when a self-tolerant light chain partner is found.

Evidence in support of a special role for light chains in the etiology of autoimmune diseases also comes from a consideration of mouse disease models. There are several types of mouse model of autoimmune disease (97). Autoimmunity can be induced by challenging animals with self-antigen in the presence of powerful adjuvants. An example is the Experimental Allergic Encephalitis (EAE) model that involves the challenge of SJL mice with spinal cord homogenate (97). Other models of autoimmune disease involve the spontaneous development of disease. This is the case with the NOD Type 1 Diabetes model and models of SLE using MRL/lpr mice and (NZB × NZM)F1 mice (97). These spontaneous models may more closely approximate human disease than the antigen challenge models.

A third kind of model relies upon genetic manipulation of animals using gene knockout and transgenic techniques. These models have been particularly important for the study of self-reactive B and T cells, and how they are deleted or otherwise controlled. Some of these models involve the use of transgenic antigen and antibody pairs (eg HEL/anti-HEL) (98). Other models use transgenic immunoglobulin chains derived from autoreactive B cells arising in autoimmune-prone mice. For example, Andrews and colleagues recently published a study exploring receptor editing in mice that carry an IGKV4-IGKJ4 anti-DNA transgene (99). Although more self-reactive cells were seen when the transgene was expressed in autoimmune-prone MRL/lpr mice, self-reactive B cells were also generated when the transgene was expressed in C57BL/6 mice (99).

This IGKV4-IGKJ4 anti-DNA transgene sequence is derived from a monoclonal antibody that was first isolated from an MRL/lpr mouse in 1987 (100, 101). In describing this and other anti-DNA antibodies, the authors acknowledged their lack of knowledge of the germline genes in MRL/lpr mice, but concluded that the mAb antibody gene sequences were relatively unmutated, based upon a consensus sequence created from both the anti-DNA and other non-DNA-specific antibodies. The apparent presence of some somatic point mutations was, however, deemed to be highly significant. In fact studies describing these antibodies stand as the first evidence for the possibility that self-reactive B cells can arise from self-tolerant B cells by the accumulation of somatic point mutations within the germinal center reaction (100, 101).

Thirty years later, our understanding of MRL/lpr germline genes is still far from complete, but comparisons can now be made between the anti-DNA antibodies and the complete repertoire of C57BL/6 IGKV genes and other murine IGKV genes. This includes sequences that are likely to be NOD IGKV germline genes, many of which are identical to MPL-derived IGKV sequences in GenBank (31). The IGKV sequence in the transgene includes 18 nucleotide differences with respect to the nearest reported IGKV gene (*IGKV4-81*) in the IMGT reference directory. The sequence is, however, much more similar to a NOD sequence reported by Henry and colleagues, differing only within the CDR3 region of the sequence (31). We believe that the many differences with respect to C57BL/6 IGKV genes are a consequence of the separate evolutionary origins of the IGKV loci of the C57BL/6 and MRL/lpr mouse strains. Based upon the haplotype analysis depicted in Figure 1, the MRL/MpJ-derived transgene appears to be of *M. m. castaneus* origin. In the absence of further information about the MRL/lpr IGK locus, there can now be no certainty regarding the presence or absence of somatic point mutations in the anti-DNA sequences reported in 1987. Only when the germline IGKV genes of MRL mice have been properly documented will it be possible to say whether or not these anti-DNA antibodies arose through an accumulation of point mutations in previously self-tolerant cells.

We believe that the autoreactivity of the light chain product of the IGKV4-IGKJ4 transgene may be the result of its *M. m. castaneus* origin, and of its association with *M. m. domesticus* and *M. m. musculus*-derived heavy chains. We also believe that *M. m. castaneus* genes may also explain the spontaneous autoreactivity that is seen in NOD and other inbred mice. The complete κ light chain locus of the NOD strain, and portions of the loci of the MRL/lpr and NZB strains, are derived from the *M. m. castaneus* sub-species of the house mouse (Figure 1). The heavy chain locus of the NOD mouse, on the other hand, comes from the *M. m. musculus* sub-species, while the MRL/lpr and NZB strains appear to carry IGH loci that are *M. m. domesticus*-derived.

The three major sub-species of the house mouse emerged from a common ancestor about 350,000 years ago (102), and it is reasonable to assume that their heavy and light chain genes co-evolved as the sub-species diverged. This co-evolution would be required to minimize self-reactivity, and to ensure that each heavy chain could successfully partner with light chains encoded by at least a subset of the IGKV genes. It appears, however, that the breeding histories of many laboratory mice have resulted in heavy and light chain gene sets that did not evolve together being found in their genomes. A few common laboratory strains, including BALB/c and 129 mice, carry matching *M. m. domesticus*-derived IGH and IGK loci, whereas others like the AEJ, C57BL/6, C57BL/10, and SJL strains carry a *M. m. domesticus*-derived IGH locus but a *M. m. musculus*-derived κ locus (Figure 1).

Not all inbred mice that have been reported to be prone to autoimmunity harbor obviously mismatched loci. For example, C57BLKS/J mice are diabetes-prone, but have heavy and light chain loci that appear to be derived from *M. m. domesticus* (103). DBA mice are used in a collagen-induced arthritis model of rheumatoid arthritis (104), and both their heavy and light chain loci also appear to be *M. m. domesticus*-derived. It is also true that not all strains that carry mismatched loci have been reported to be susceptible to autoimmunity. An example is the RF/J strain, which appears to have a *M. m. domesticus* IGH locus and a *M. m. castaneus* IGK locus. However, it is striking how many models of autoimmunity involve mismatched heavy and light chain gene loci. In addition to the NOD, MRL/lpr, and NZB models, SJL mice that are used in the EAE model of multiple sclerosis (97) appear to have a *M. m. musculus* IGH locus and a *M. m. domesticus* IGK locus. A model of autoreactivity to matrix collagen uses a C57BL/6-derived IGKV3 transgene in C57BL/6 hosts (105). In this strain, the IGH locus seems to be *M. m. musculus*-derived, while the IGK locus is *M. m. domesticus*-derived. Hybrid (129 × C57BL/6) mice spontaneously develop an SLE-like condition (106), and these mice express *M. m. domesticus* heavy chains in association with both *M. m. domesticus* and *M. m. musculus*-derived κ chains. Finally, the BXSB mouse spontaneously develops lupus-like pathology (107). SNP analysis suggests that it has a *M. m. domesticus* IGH locus and a *M. m. musculus* κ locus (Figure 1).

For over 30 years, mouse models have provided profound insights into the nature of autoreactivity and self-tolerance. It may be though that an ignorance of the makeup of the immunoglobulin gene loci has kept hidden a key genetic contributor to autoimmunity. To determine if this may be the case, the repertoires of laboratory mice will need to be compared to repertoires generated in animals in which the heavy and light chain loci and all critical regulatory elements, as well as self-antigens, are all derived from the same sub-species of the house mouse. The immunoglobulin genes of the different strains will also need to be properly characterized. It is possible that this may reveal that the apparently matched loci of some autoimmune-prone strains are derived from disparate sources. SNP analysis at present characterizes mouse strains with respect to the three major sub-species of the house mouse, but other minor sub-species may also have contributed genes to the laboratory mouse. We believe that such a focus on heavy and light chain pairings, in mouse models and in human studies, may help explain some of the mysteries that still surround autoimmune diseases.

## Author contributions

All authors listed have made a substantial, direct and intellectual contribution to the work, and approved it for publication.

### Conflict of interest statement

The authors declare that the research was conducted in the absence of any commercial or financial relationships that could be construed as a potential conflict of interest. The handling editor declared a past co-authorship with one of the authors AC.
