# Supplementary material for: Immunoglobulin Light Chain Gene Rearrangements, Receptor Editing and the Development of a Self-Tolerant Antibody Repertoire
Source: Front Immunol. 2018 Oct 8;9:2249. doi: 10.3389/fimmu.2018.02249 (PMC6186787; doi:10.3389/fimmu.2018.02249)
Supplement: Supplementary file 1 [file Data_Sheet_1.docx]

**Figure S1**: A phylogeny depicting the phylogenetic relationships of mouse (C57BL/6) and human IGHV (blue), IGLV (red), and IGKV (black) gene segment sequences (coding bases only), distinguished by open (mouse) and filled (human) symbols. The topology of the tree reveals species-specific expansions and contractions of gene subfamilies; for example, the contraction of IGLV genes and large expansion of family IGHV1 genes in mouse. To construct the tree, sequences for *01 of human IGHV, IGKV, and IGLV, as well as mouse IGHV and IGKV were first downloaded from the IMmunoGeneTics Information System (IMGT; www.imgt.org/vquest/refseqh.html); for mouse, only sequences from the C57BL/6 strain were used. In addition, mouse (C57BL/6) IGLV sequences were extracted from the mm10 genome reference assembly. A multi-sequence alignment, involving 287 nucleotide sequences in total, was performed using MUSCLE (1) within the MEGA7 software package (2). Using MEGA7, the phylogeny was constructed using the Neighbor-Joining method (3), and evolutionary distances were computed using the Maximum Composite Likelihood (4); gaps and missing data were not considered.

REFERENCES:

1. Saitou N. and Nei M. (1987). The neighbor-joining method: A new method for reconstructing phylogenetic trees. Molecular Biology and Evolution 4:406-425.2.
2. Tamura K., Nei M., and Kumar S. (2004). Prospects for inferring very large phylogenies by using the neighbor-joining method. Proceedings of the National Academy of Sciences (USA) 101:11030-11035.3.
3. Kumar S., Stecher G., and Tamura K. (2016). MEGA7: Molecular Evolutionary Genetics Analysis version 7.0 for bigger datasets.Molecular Biology and Evolution 33:1870-1874.
4. Edgar RC. MUSCLE: a multiple sequence alignment method with reduced time and space complexity. BMC Bioinformatics. 2004 5:113.
